# Infectiological, functional, and radiographic outcome after revision for prosthetic hip infection according to a strict algorithm: 22 one-stage and 50 two-stage revisions with a mean follow-up time of 5 (2–17) years

**DOI:** 10.3109/17453674.2010.548025

**Published:** 2011-02-10

**Authors:** F Harald R De Man, Parham Sendi, Werner Zimmerli, Thomas B Maurer, Peter E Ochsner, Thomas Ilchmann

**Affiliations:** ^1^Department of Orthopaedics, Sint Maartenskliniek, Nijmegen, the Netherlands; ^2^Clinic of Orthopaedic Surgery, Kantonsspital, and the Infectious Diseases Unit, Basel University Medical Clinic, Liestal, Switzerland

## Abstract

**Background and purpose:**

Successful treatment of prosthetic hip joint infection (PI) means elimination of infection and restored hip function. However, functional outcome is rarely studied. We analyzed the outcome of the strict use of a treatment algorithm for PI.

**Patients and methods:**

The study groups included 22 hips with 1-stage exchange for PI (group 1), 22 matched hips revised for aseptic loosening (controls), and 50 hips with 2-stage exchange (group 2). Relapse of infection, Harris hip score (HHS), limping, use of crutches, reoperations, complications, and radiographic changes were compared between the groups.

**Results:**

There was 1 relapse of infection, which occurred in group 2. In group 1, the mean HHS was 84; 4 of 19 patients were limping and 2 required 2 crutches, which was similar to the control results. In group 2, scores were lower and complication rates higher. The use of a Burch-Schneider ring and the presence of a deficient trochanter impaired function. There were no differences in radiographic outcome between the groups.

**Interpretation:**

With the algorithm used, infection can be cured with high reliability. With a 1-stage procedure, mobility is maintained. After 2-stage procedures, function was impaired due to there being more previous surgery and more serious infection.

Infections associated with prosthetic joints cause significant morbidity and account for a substantial proportion of healthcare expenditure (Bozic and Ries 2005). The management of infection associated with prosthetic joints is poorly standardized because of the varied clinical presentations and the lack of data from randomized, controlled trials. We have recently published a treatment algorithm that was developed at the Kantonsspital Liestal over the past 25 years (Zimmerli et al. 2004). Adherence to this treatment concept has shown a success rate of 85–100% in curing infection (Giulieri et al. 2004, Sendi et al. 2006).

Successful treatment of prosthetic hip joint infection (PI) consists not only of eliminating the infection but also of restoring patient mobility, which is important for patient satisfaction (Britton et al. 1997). Even so, functional and radiographic outcome has been mainly investigated in aseptic revisions (Saleh et al. 2003). Ideally, the evaluation of any treatment algorithm for PI should be from a multidisciplinary perspective, including infectiological, radiographic, and functional outcome.

Our surgical technique and choice of components are entirely dictated by the quality of soft tissue and bone. In our view, the functional outcome after 1-stage exchange for PI should therefore be similar to that for aseptic reasons. After 2-stage exchange, this outcome is expected to be worse because this intervention is commonly performed in patients with more severe infections, requiring a more complex surgical procedure. Moreover, in revision hip surgery and especially in cases with PI, patient and surgical factors (e.g. femoral osteotomy and components) are highly variable because of the different quality of soft tissue and bone in individual cases. Thus, when reporting on functional outcome in cases of PI, the presence of such factors should be acknowledged and their possible influence on outcome analyzed.

Because elimination of the infection is a prerequisite for a good functional outcome, we first wanted to confirm that our treatment algorithm is associated with successful infectiological outcome as reported in previous studies (Giulieri et al. 2004, Sendi et al. 2006). Since these reports were published, 37 additional hips have been treated accordingly and could be included for analysis of infectiological outcome. Secondly, we wanted to determine whether functional and radiographic outcome, aseptic revision, and complication rate would be different (1) after 1-stage exchange for PI as compared to 1-stage aseptic revisions, and (2) after 1-stage exchange for PI as compared to 2-stage exchange. We hypothesized that all outcomes would be similar in 1-stage groups and lower after 2-stage exchange. Thirdly, we wanted to determine whether functional outcome was affected by various pre-defined surgery-related parameters.

## Patients and methods

### Population

We retrospectively analyzed all patients who had been treated with exchange of a THA, because of PI, at our center between 1985 and 2004. We formed 3 groups. The “1-stage exchange due to PI” group (group 1), a matched aseptic control group (Control), and the “2-stage exchange” group (group 2) who were analyzed separately. Functional and radiographic parameters, rates of revision, and complications were compared between controls and group 1, and between group 1 and group 2. A transfemoral osteotomy, a deficient greater trochanter, a 2-stage revision, a history of multiple cup or stem revision, a BS-ring, or a Wagner stem were identified to possibly lead to a lower HHS, and/or more limping or use of support. In order to analyze whether or not these variables independently influenced functional outcome all hips with an event-free follow-up were pooled in one single study group and variables were analyzed separately.

**Figure 1.**
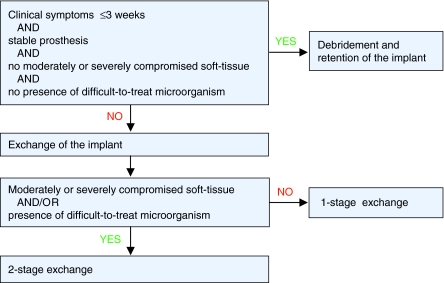
The algorithm showing decision making for a 1-stage or 2-stage revision.

To establish the diagnosis of PI, the presence of a sinus tract or the growth of the same microorganism in at least 2 cultures, or inflammation consistent with infection on histopathological examination was required (Giulieri et al. 2004, Zappe et al. 2008). Hips with a PI were only included if treatment was strictly according to the algorithm and an exchange of the implant had been done (Zimmerli et al. 2004) (Figure 1). Antimicrobial compounds were selected as described previously (Zimmerli et al. 2004) and typically administered for 8–12 weeks. Hips were excluded when results were not available 2 years after the index operation, or if they were lost to follow-up or when documentation was poor.

**Figure 2.**
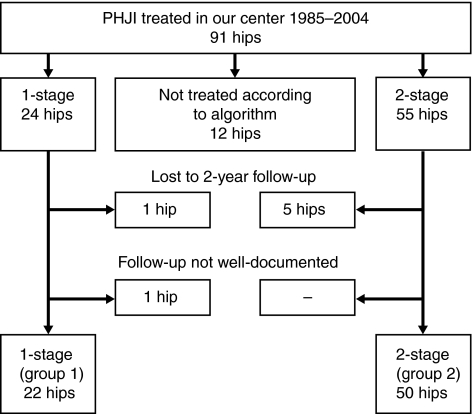
Flow of hips included in the study. Exclusion of 19 of 91 hips that did not fulfill the inclusion criteria.

91 hips were treated with a 1-stage exchange or a 2-stage exchange because of PI. 12 hips were considered not to have been treated according to the algorithm and were excluded: in 2 hips the infection was detected after death, in 2 hips treatment of infection was declined, in 4 hips components remained in site despite the presence of severe soft tissue damage because the operation risk due to co-morbidities was inordinately high and in 4 hips the infection was unknown during revision and therefore treatment started too late. In group 1, 1 hip was excluded due to loss of 2 year follow-up and another hip because follow-up was not well documented, resulting in 22 selected hips (21 patients) for final inclusion. These patients were matched with 22 patients (controls) who were selected from 474 consecutive aseptic revision procedures. Matching was performed—in decreasing order of importance—for previous surgery on trochanter, number of revisions of the cup, number of revisions of the stem, type of implant, use of transfemoral osteotomy, Charnley score, duration of follow-up, age, and sex. In group 2, 5 hips were excluded all because of loss of 2 year follow-up resulting in 50 selected hips (48 patients) for final inclusion (Figure 2), of which 34 hips had severely damaged soft tissues with a sinus and/or abcess formation.

12 of the 22 hips in group 1 (11 patients) and 39 of the 50 hips in group 2 (37 patients) had been referred. The referred cases had had more surgery before referral and qualified less often for a 1-stage exchange compared to the non-referred patients.

Staphylococci were the most commonly involved pathogens (40 hips) followed by *Streptococcus* spp. (11 hips) and gram-negative rods (8 hips). In 17 hips, the isolates were classified as difficult to treat, and included *Pseudomonas aeruginosa,* rifampin-resistant staphylococci, *Enterococcus* spp, MRSA, small-colony variants of *S. aureus,* and *Abiotrophia adiacens,* requiring a 2-stage exchange in these cases. In 10 hips the infection was polymicrobial, and in 3 hips no pathogen could be cultured.

Patients were followed up after 6 weeks, 3 months, 6 months, 1 year, 2 years, and every 5 years thereafter.

The mean clinical follow-up was 3.8 years (SD 2.2) for both group 1 and the control group, and 4.9 years (SD 3.6) for group 2. 19 of the 22 patients in group 1, all 22 in the control group, and 43 of the 50 hips in group 2 had an event-free survival for ≥ 2 years and qualified for functional outcome analysis.

### Operative technique

**Figure 3.**
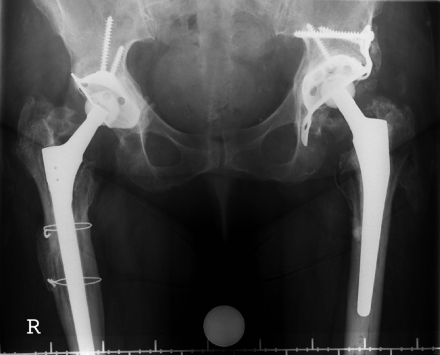
A 59-year-old woman with bilateral PI following *Staphylococcus aureus* sepsis, 2 years after bilateral 1-stage exchange. On the right side: reconstruction with a Müller reinforcement ring for a type-1 defect, and a long Wagner stem by transfemoral approach. On the left side: a reconstruction with a Burch-Schneider ring to bridge a type-2B defect. A cemented Virtec stem with transgluteal approach was implanted. At the 2-year follow-up, the HHS (bilateral) was 97, there was a slight limp, there were no radiographic signs of loosening, the transfemoral osteotomy was healed, and the infection was cured.

The index operation was defined as the reimplantation procedure done at our center. Bone defects on the latest preoperative radiographs were classified according to Paprosky (Paprosky et al. 1994, Valle and Paprosky 2003) (Table 1). Patients were operated in the supine position with a straight lateral approach. In 34 hips, this was combined with a transfemoral osteotomy (Wagner and Wagner 1999) (Figure 3). In the infected cases, after removal of the components a thorough debridement was performed—taking care to preserve bone and soft tissue. In all groups, the choice of a particular implant was dictated by the classification of bone defects irrespective of the presence of infection. For reconstruction of the acetabulum, an uncemented cup (SL-Müller), a reinforcement ring (Müller), or a Burch-Schneider (BS) reinforcement ring (all Zimmer, Warsaw, IN) were used. Morselized autograft were added for small defects, and/or slices of allograft for larger defects. For reconstruction of the femur, an uncemented titanium stem (Wagner SL) or a cemented stem (Müller straight stem; CDH, Virtec) with gentamicin cement (Palacos) was used (all Zimmer, Warsaw, IN). In 2-stage procedures, reimplantation was performed after 3–5 weeks (damaged soft tissue) or after 6–8 weeks (difficult-to-treat bacteria). In group 2, a Burch-Schneider (BS) reinforcement ring was needed more often (Table 2).

**Table 1.** Patient characteristics prior to the index operation

		Matched **^a^**			
	Control	p-value	Group 1	p-value	Group 2
Total hip arthroplasties (patients)	22 (22)		22 (21)		50 (48)
Age, median	67	0.4 **^b^**	69	0.2 **^b^**	70
range	53–84		48–88		40–88
Male	15	0.2 **^c^**	10	0.1 **^c^**	29
Charnley classification		0.9 **^d^**		0.15 **^d^**	
A	12		14		31
B1	1		1		0
B2	9		7		17
C	0		0		2
Previous surgery performed	2	1.0 **^c^**	3	< 0.001 **^c^**	39
on trochanter	2	1.0 **^d^**	2	0.4 **^d^**	17
on cup, for any reason	1	0.3 **^d^**	0	0.002 **^d^**	23
on stem, for any reason	2	1 **^d^**	2	0.01 **^d^**	25
debridement(s)	0	1 **^d^**	0	< 0.001 **^d^**	70 **^e^**
Girdlestone/spacer	–		–		4/2
Proprosky classification cup		0.5 **^d^**	**^f^**	0.004 **^d^**	**^f^**
1	13		15		17
2A	3		1		5
2B	2		2		5
2C	2		2		12
3A	2		1		6
3B	0		0		4
Proprosky classification stem		0.6 **^d^**	**^f^**	0.2 **^d^**	**^f^**
I	0		1		1
II	17		16		19
IIIA	4		3		20
IIIB	1		1		2
IV	0		0		7
Deficient greater trochanter	1	1 **^c^**	1	0.05 **^c^**	12

**^a^** Control and group 1 were matched for all listed variables.

**^b^** Student t-test

**^c^** Chi-square test.

**^d^** Mann-Whitney U-test.

**^e^** 16 hips received ≥ 2 (range 2–10) debridements.

**^f^** One preoperative radiograph was not available.

**Table 2.** Surgical procedures and specifics at the index operation

		Matched **^a^**			
	Control		Group 1		Group 2
Total hip arthroplasties (patients)	22 (22)	p-value	22 (21)	p-value	50 (48)
No osteotomy	14	0.5 **^b^**	15	0.2 **^b^**	22
Transfemoral osteotomy	7	0.5	5	0.1	22
Greater trochanter osteotomy	1	1	1	0.5	1
Approach via nonunion in greater trochanter	0	0.9	1	0.2	5
Spacer	–	–	–	–	29
Acetabular component					**^c^**
SL uncemented cup	2	1 **^b^**	2	0.6 **^b^**	2
Müller reinforcement ring	16	1	16	0.08	24
Burch-Schneider reinforcement ring	4	1	4	0.03	23
Femoral component		1 **^b^**		0.8 **^b^**	**^c^**
uncemented	11		11		26
cemented	11		11		23
Acetabular graft		0.2 **^d^**		0.9 **^d^**	**^c^**
no graft	5		9		27
autograft, morselized	4		10		9
allograft, blocks > 2 cm or slices	2		3		10
auto- and allograft	1		0		3

**^a^** Control and group 1 were matched for all the variables listed.

**^b^** Chi-square test.

**^c^** In group 2, one patient died prior to reimplantation (see also complications, Table 4).

**^d^** Mann-Whitney U-test.

### Infectiological, clinical, and radiographic evaluation

The infection was classified as “cured” when there were no signs of infection 2 years or more after implantation (Giulieri et al. 2004). In case of death for an unrelated cause before the 2-year follow-up, infection was classified as “probably cured”. Relapse was defined as an infection with the same pathogen, and reinfection was defined as an infection with a different microorganism. Functional outcome parameters were the Harris hip score (HHS), the presence of limping, and the use of a support, and they were only recorded in patients with an event-free follow-up at 2 years (no revision and no death). This time point was chosen because after 2 years, results decline due to patient-related factors (Riede et al. 2007). Complications and reoperations were recorded for all the hips included.

For radiographic follow-up, AP radiographs of pelvis and femur faux–profiles views were scrutinized for signs of loosening according to Gill et al. (1998) for the acetabulum and according to Harris (1982) for the femur. Migration and subsidence were measured (Callaghan et al. 1985). In case of osteotomy or bone lesions, images were screened for signs of non-union. All radiographic variables were obtained from the latest available radiograph. Endpoints for both clinical and radiographical follow-up were re-revision or death.

### Statistics

Statistical comparisons for patient characteristics, surgical specifics, and functional, radiographic, and microbiological outcomes were conducted with chi-square and Student's t-test. For non-parametric data, the Mann-Whitney U test was used. For all 84 hips with an event-free follow-up, multiple univariate regression analysis (ANOVA) was performed to determine whether surgical parameters lead to lower functional outcome(s). Calculations were done with SPSS software version 15.0 and statistical significance was set at p < 0.05.

### Ethics

Informed consent was obtained from all patients. The study design was approved by the local ethics committee of the hospital district in which the study was conducted (01-05-2006; WN 420).

## Results

### Infectiological outcome

In group 1, there was no relapse of infection but there was 1 case of reinfection with another pathogen. In group 2, there was 1 relapse of infection (Table 3).

**Table 3.** Infectiological, functional, and radiographic outcome, and number of revisions

	Control	p-value	Group 1	p-value	Group 2
Infectiological outcome				0.5 **^a^**	
total hip arthroplasties	22		22		50
cured	–	–	19		46
probably cured	–	–	2		1
relapse of infection with same pathogen	–	–	0		1
reinfection with different pathogen	0	0.2 **^b^**	1		0
unknown	–	–	0		2
Functional outcome at 2-year follow-up					
total hip arthroplasty **^c^**	22		19		43
mean HHS (SD)	85 (16)	0.9 **^d^**	84 (17)	0.3 **^d^**	80 (18)
mean HHS with/without spacer	–	–	–	–	80/78
limping		0.5 **^a^**		0.5 **^a^**	
none or slight	19		15		30
moderate or severe	3		4		13
walking		0.2 **^a^**		0.5 **^a^**	
without support	15		9		18
with one cane/crutch	7		8		13
with two canes/crutches	0		2		11
unable to walk/use of wheelchair	0		0		1
Radiographic outcome at last follow-up ^**e**^					
total hip arthroplasties	22		20 **^f^**		47 **^f^**
mean follow-up in years (SD)	4.8 (2.8)	0.05 **^d^**	3.3 (2.4)	0.5 **^d^**	4.0 (3.7)
definitive loosening of stem (revised)	1 (1)	0.5 **^b^**	2 (2)	0.9 **^b^**	4 (2)
definitive loosening of cup (revised)	1 (0)	0.2 **^b^**	0	0.1 **^b^**	3 (1)
stem subsidence > 5 mm (revised)	0	–	0	0.6 **^b^**	2 (0)

**^a^** Mann-Whitney U-test.

**^b^** Chi-square test.

**^c^** Only hips with an event-free survival for ≥ 2 years were included in functional outcome analyses.

**^d^** Student t-test.

**^e^** Statistical analysis was performed with endpoints definitive component loosening and stem subsidence > 5 mm.

**^f^** For 2 hips, in group 1 and group 2, radiographs were not available since the patients died prior to the follow-up examinations. Also, postoperative radiographs were not available for another hip in group 2.

### Functional and radiographic outcome, revisions, and complications

#### Control vs. group 1

We did not find any statistically significant differences in the functional parameters HHS, limping, and use of support—or in radiographic parameters—between the 2 groups. Definitive loosening of the stem with subsequent revision occurred in 2 hips in group 1 and in 1 hip in the control group (Table 3). 1 cup in the control group showed signs of definitive loosening but the hip score was 100 and revision was not performed. All osteotomies and the pre-existing nonunion of the greater trochanter healed successfully. 1 iatrogenic fracture of the greater trochanter that occurred in group 1 developed a nonunion. The total number of postoperative complications and re-interventions was similar in both groups (Table 4).

**Table 4.** Number of complications associated with the index operation

	Control	p-value	Group 1	p-value	Group 2
Total hip arthroplasties	22 (22)		22 (21)		50 (48)
Surgical complications					
fracture, greater trochanter	3		1		1
fracture/fissure, proximal femur	1		3		2
Post-surgical complications					
requiring reoperation					
hematoma	2		1		17
wound infection	0		0		1
pin tract infection	–		–		1
requiring closed reduction					
dislocation of spacer	–		–		1
dislocation of hip	0		0		6
total reinterventions	2	0.5 **^a^**	1	0.004 **^a^**	26
Total (post-) surgical complications	6	0.9 **^a^**	5	0.03 **^a^**	29
Non-surgical complications					
during hospitalization					
thrombosis/emboli	0		0		2
miscellaneous	1		3		4
early death	0		0		2 **^b^**

**^a^** Mann-Whitney U-test.

**^b^** Death due to heart failure and pneumonia, one month after the index operation.

#### Group 1 vs. group 2

Comparing both groups, all functional outcomes were lower in group 2 but the differences were not statistically significant (Table 3). Also, radiographic results were not significally different between groups 1 and 2. In group 2, 4 stems showed signs of definitive loosening and 2 of those were revised; the other 2 stems showed subsidence of 4 mm and 28 mm, but they remained stable and patient co-morbidity did not allow intervention. Also, 3 cups were definitively loose: 1 of them (a Müller reinforcement ring) was revised and the other 2 (BS-rings) did not cause symptoms (Table 3). 1 of the transfemoral osteotomies and the only trochanteric osteotomy developed a nonunion. 2 of the 5 pre-existing nonunions of the greater trochanter persisted. All intraoperative fractures of the proximal femur healed uneventfully. Group 2 had a higher incidence of (post-) surgical complications, mainly due to more postoperative hematomas and dislocations, and had more re-interventions (Table 4).

### Surgery-related parameters and functional outcome

The use of a transfemoral osteotomy was not associated with lower functional outcome (p = 0.1 for HHS; p = 0.2 for limping; p = 0.9 for use of support). A deficient greater trochanter (p = 0.01 for limping; p = 0.05 for use of support), the use of a BS-ring (p = 0.03 for limping), a trochanteric osteotomy (p = 0.05 for use of support), and a history of multiple cup revisions (p = 0.06 for use of support) were associated with lower functional outcome.

## Discussion

A commonly proposed treatment strategy for PI is a 2-stage procedure with up to a 6-month interval between surgeries (McDonald et al. 1989, Berry et al. 1991, Colyer and Capello 1994, Lieberman et al. 1994, Nestor et al. 1994, Garvin and Hanssen 1995, Lai et al. 1996, Wang and Chen 1997). However, the use of a 1-stage exchange provides several advantages such as lower perioperative morbidity, a shorter hospital stay, lower costs, and earlier rehabilitation (Buchholz et al. 1981, Wroblewski 1986, Raut et al. 1994, 1995, 1996, Ure et al. 1998, Bozic and Ries 2005). Yet, the results of some reports indicate the possibility of a higher relapse rate (Raut et al. 1994, 1995). This implies that only selected patients may benefit from a 1-stage procedure. With this line of reasoning, we have established an algorithm that selects patients for either a 1-stage exchange or a 2-stage exchange according to well-defined criteria (Zimmerli et al. 2004). In agreement with our previous studies (Giulieri et al. 2004, Sendi et al. 2006), we found a very low relapse rate of infection in the patients in the present study. In the study, we focused on functional outcome.

This study had several limitations. First, the number of patients was small. On the other hand, our analysis included only hips that were treated according to a strict protocol with a multidisciplinary approach, and there was almost no relapse. Inclusion of the simultaneous analysis of functional, radiographic, and infectiological parameters is crucial when judging the outcome of a treatment algorithm for PI. Also, we applied strict methodological criteria to functional outcome evaluation by only including hips that had an event-free follow-up of at least 2 years. Given these study constraints, it is conceivable that the sizes of the groups were relatively small. We cannot exclude the possibility that the statistically insignificant differences we found between infectious and non-infectious hips might have become significant in a larger series. However, in our view it is not feasible to perform a study with the high numbers of hips required from a statistical point of view that would also include a standardized treatment and a multidisciplinary follow-up as performed in our study. We believe that our analysis was conducted on a solid study population and reflects clinical reality. Secondly, the preoperative functional scores were not available for the hips included in the study. This partially limits the interpretation of the 2-year HHS. However, many patients had had previous surgery and complaints due to infection; thus, such preoperative scores would not allow any reliable comparison with the postoperative state. Thirdly, we used several different implants. As in any revision surgery, the variability in quality of bone is often substantial, requiring an individual choice of implant; our algorithm was not bound to any preferred reconstruction technique. Finally, functional outcome was based on only 3 variables (i.e. HHS, limping, and walking support). We focused on simple variables, however, to allow comparison between groups, and between our results and those reported in the literature.

HHS was almost identical in the 1-stage group and in the control group (mean HHS of 84 and 85). Moreover, the values were higher when compared to a report of 31 one-stage procedures for infection, in which the mean HHS was 75 after 3.5 years (Tsukayama et al. 1996). In a recent meta-analysis of 24 studies analyzing mainly aseptic revisions, the mean HHS was 82 after a mean follow-up of 4 years (Saleh et al. 2003). The high functional scores after 1-stage exchange can be partly explained by the design of the algorithm, because it selects less complex cases for such procedures.

This means that the algorithm selects more complex cases for a 2-stage exchange. These cases had moderately or severely damaged soft tissue (i.e. sinus tract, multiple previous surgeries), or involved a difficult-to-treat microorganism. Since a 2-stage exchange is only performed in cases of infection, there was no matched control group available. Even so, the functional scores of the 2-stage group were good (mean HHS of 80), although they were lower than those of the 1-stage group (mean HHS of 84). A difference of 4 points may be clinically important (Hoeksma et al. 2003) and such a discrepancy is not surprising considering the preoperative conditions—including multiple surgeries, more serious infections, limited bone stock, and deficiency of the greater trochanter. Consequently, the surgical procedure becomes more complex. The Harris hip scores in our study (80–85) are similar to those in the literature, where the scores reported have ranged from 72 to 91 (Lai et al. 1996, Haddad et al. 2000).

A transfemoral osteotomy, an important feature of our surgical technique, was not associated with poor functional outcome. With this technique (Wagner and Wagner 1999), safe removal of the components and a thorough debridement can be achieved. None of the osteotomies were associated with a periprosthetic fracture; the only relapse of infection occurred in a case of osteotomy but appeared not to be related to this technique, and another developed a nonunion. An important aspect of our treatment strategy is that we strive to preserve bone and soft tissue as much as possible in order to optimize the functional result. This contrasts with other surgical regimens where infected tissue is radically debrided or even resected (Friesecke and Wodtke 2008, Wodtke and Lohr 2008). In our study, infections were eliminated in almost all cases without the need for aggressive removal of bone and soft tissue.

We found that a trochanter deficiency and the use of a BS-ring were associated with an increased risk of limping. Whereas the occurrence of limping may be explained by the presence of a deficient trochanter with impairment of the abductor apparatus, this appears to be less evident with the use of a BS-ring. It has been proposed that the exposure of the ileum may damage the anterior part of the gluteus medius muscle or the superior gluteal nerve (Possai et al. 1996, Perez et al. 2004, Ikeuchi et al. 2006). However, this finding may also be due to a confounding factor, considering that a BS-ring was implanted in 6 of 14 patients with a trochanter deficiency. The uncemented Wagner SL stem was associated with a good functional outcome and in contrast to previous reports (Hartwig et al. 1996, Kolstad et al. 1996, Bircher et al. 2001), stem subsidence was uncommon. The incidence of loosening of acetabular and femoral components was low and similar to that for identical implants used in aseptic revision surgery with equal length of follow-up (Haentjens et al. 1986, Korovessis et al. 1992, Peters et al. 1995, Ilchmann et al. 2006).

Considering the criteria in our algorithm (including the causative pathogen), we found that some of the referred cases should have been treated with a 1-stage exchange or a 2-stage exchange a priori, instead of having inadequate trials of debridement with implant retention or 1-stage exchange, respectively. Initial treatment failure with prosthetic infections increases the probability of a requirement for a more complex procedure (2-stage exchange) with a greater burden for the patients, and might therefore end in an inferior functional outcome.

Our findings support the idea of using well-defined criteria in order to select an optimal surgical strategy for patients with prosthetic infections. In our experience, the algorithm used here provides such criteria, and is therefore helpful in the surgical decision making. In this way, relapse of infection and impairment of the functional outcome can be avoided.
